# The 4P telehealth business framework for Iran

**DOI:** 10.1186/s12911-022-02011-4

**Published:** 2022-10-09

**Authors:** Farnia Velayati, Haleh Ayatollahi, Morteza Hemmat, Reza Dehghan

**Affiliations:** 1 Telemedicine Research Center, National Research Institute of Tuberculosis and Lung Diseases (NRITLD), Shahid Beheshti University of Medical Sciences, Tehran, Iran; 2 Department of Health Information Management, School of Health Management and Information Sciences, Iran University of Medical Sciences, Tehran, Iran; 3 Health Management and Economics Research Center, Health Management Research Institute, Iran University of Medical Sciences, Tehran, Iran No. 4, Rashid Yasemi St, Vali-Asr St, 1996713883; 4 Saveh University of Medical Sciences, Saveh, Iran; 5 Department of Health Entrepreneurship, Virtual University of Medical Sciences, Tehran, Iran

**Keywords:** Health service marketing, Business, Telemedicine, Telehealth

## Abstract

**Background:**

Telehealth services can utilize various information technologies and improve quality and efficiency of healthcare delivery by facilitating education, treatment, follow-up, and decision-making. However, these services are not always commercialized, and in case of commercialization, there is no guarantee for their long-term sustainability in market. Therefore, business models and frameworks are used as part of commercialization processes to identify a set of factors affecting the sustainability, effectiveness, and key business activities. The current study aimed to develop a telehealth business framework for Iran.

**Methods:**

This research was conducted in 2021, and a mixed-methods approach was used for data collection. Initially, a telehealth business framework was developed based on the findings derived from a systematic review and a qualitative research. The proposed framework was then reviewed by an expert panel (n = 9) in which the participants had at least three years of work experience in telehealth. Finally, the framework was validated using the Delphi method (three rounds).

**Results:**

The expert panel believed that some components such as partners’ expertise, required capital and financial resources, research and analysis, marketing and branding, tax, product registration, and marketing at scientific congresses and science and technology exhibitions needed to be added to the framework. In the Delphi study, 68 out of 74 components proposed in the initial framework were approved across four major dimensions; namely, prerequisites, production, payments and costs, and post-production services.

**Conclusions:**

It seems that the developed framework can facilitate commercializing telehealth technologies and developing business plans. In addition, telehealth start-ups can use this framework and its various components in a competitive market to be more successful in their businesses. However, it is still critical to evaluate the effectiveness of the framework in practice and in relation to the commercialization of telehealth technologies.

## Introduction

It is more than two decades that patients' needs for receiving continuous healthcare services have been addressed by using telehealth technology, and it is rapidly expanding across the world [1, 2]. Telehealth encompasses multiple facets of healthcare and covers a broad range of information and communication technologies used to deliver and support health care services [3–5]. It is notable that the terms telemedicine and telehealth have been used interchangeably in several studies over the years [3, 6, 7]. However, in the current study we use the term “telehealth”, as compared to telemedicine, it refers to a broader scope of remote health care services.

Telehealth technologies are regarded as a means for enhancing the quality and efficiency of health care services mainly by facilitating education, treatment, follow-up, and decision-making [8, 9]. In addition, these technologies have the potential to reduce the number of face-to-face visits, increase access to health care services, provide better resource utilization, reduce costs, and improve communication across primary, secondary and tertiary care settings [8–10].

However, there is no guarantee that transitioning telehealth technologies from the experimental stage to the operational and commercial stage results in long-term and widespread success [11]. Some of these technologies never reach the market, and according to the literature, 75% of businesses and projects fail due to the several technical and non-technical factors. This figure even rises to 90% in developing countries [12]. For example, in Iran, the commercialization of telehealth technology has been hindered by several factors such as physician resistance and insufficient funding to develop and support telehealth systems [13]. Other difficulties associated with the use of telehealth technology in Iran include inadequacy of the users’ knowledge, limited physical and financial resources and facilities, insufficient cooperation among the stakeholders, insurance and reimbursement issues, and a lack of transparency over the property rights and basic infrastructure costs [14, 15]. The coordination of clinical activities among a diverse group of healthcare providers is also difficult when providing telehealth services [16, 17].

The customers of telehealth services are various and include a wide range of stakeholders, patients, patients’ attendance, healthcare providers, hospital staff and management, application developers and managers, insurance companies, and information technology providers [6, 18–21]. Moreover, the concept of “customer” is considered distinct from “consumer”, as sometimes an institution or organization purchases the technology as a “customer”, but another person, e.g., a patient or an organization uses the services as a “consumer” [19]. Therefore, to run a successful telehealth business, a holistic approach is required to address all issues related to technology, organizational structures, change management, economic feasibility, social impact, users’ perceptions, usability issues, evaluation, legislation, and governance [20, 21]. This approach has been presented in the business models (BM) and frameworks to facilitate identifying a set of components that influences the long term sustainability of innovations, such as telehealth technology in the market [22]. A business model is a high-level conceptual description of a business that shows how a company creates, delivers, and captures value for the customers as well as itself [23].

According to a systematic review conducted by Velayati et al., although there are a number of business models and frameworks that are used in the field of telehealth, they may not cover all dimensions or components of a telehealth business [24]. Therefore, developing new models and frameworks with diverse components is suggested to cover different aspects of telehealth technology [24–26]. Some of these components are created value, key activities, key resources, key partners, licenses and permissions, product pricing, product revenue, product marketing, support services, and obtaining feedback [27].

Given that each country has distinct organizational structures, insurance policies, information technology infrastructure, economic status, culture, and values, components affecting a telehealth business might be different, and the current business models and frameworks may not be useful for various purposes. Therefore, developing a telehealth business framework can help to obtain a more comprehensive understanding of the components influencing the successful telehealth technology commercialization. The current study aimed to develop a telehealth business framework for Iran.

## Methods

This research was conducted in 2021, and a mixed-methods approach was used for data collection. Before conducting the research, the ethical approval was obtained from the ethics committee of Iran University of Medical Sciences (IR.IUMS.REC.1397.1328). Initially, a systematic review was conducted, and the telehealth business models and frameworks were reviewed [24]. Then, a qualitative study were conducted, and people who were experts in medical informatics, health information management, health entrepreneurship, and telehealth business were interviewed to identify the main components of a telehealth business framework [27]. The findings derived from the first and second phases of the research were presented as a proposed telehealth business framework to an expert panel. After applying the experts’ comments to the framework, the Delphi study (three rounds) was conducted to validate the proposed framework.

### Research participants

The participants of the expert panel (n = 9) were among the people who had taken part in the second phase of the study. In the Delphi study (three rounds), 65 experts who had a background in medical informatics, health information management, health entrepreneurship, and telehealth business were found eligible and invited to take part in the study. In total, 21 experts participated in the first round, and 14 individuals took part in the second and third rounds.

### Research instrument

The data were collected between April and September 2021. Before conducting the expert panel, the proposed telehealth business framework was sent to the experts. In the expert panel, the participants were asked about the suitability of the dimensions and components of the framework, and based on their comments, changes were made to the framework simultaneously.

Then, the Delphi study was conducted using a five-point Likert scale online questionnaire. The questionnaire included the components which were finalized in the expert panel. In the first round of the Delphi study, the questionnaire included the components of telehealth business prerequisites (11 components), production, product delivery, and service delivery (34 components), payments, costs, and revenue generation of the product or service (15 components), and post-production services (14 components). In the second round of the Delphi study, the questionnaire included 13 components, and in the third round, the participants were asked about 5 remaining components that did not reach a consensus in the previous rounds. The time period between each Delphi round was three weeks. The face and content validity of the first questionnaire were assessed by three experts in medical informatics, health information management, and health entrepreneurship.

### Data analysis

The data collected from the expert panel were analyzed and described narratively. The results of the Delphi study were analyzed using descriptive statistics. In total, if 75% of the participants or more selected the first two options of the questionnaire (very important or important) for each component, and the mean value was more than 3.75, the component would be included in the final framework. If between 50 and 75% of the participants chose the first two options, or the mean value was between 2.5 and 3.75, the item would be asked again in the second round of the Delphi study. If less than 50% of the participants selected the first two options, or the mean value was less than 2.5, the relevant component would be removed from the final framework. This process was repeated for the second and third rounds of the Delphi study, too.

## Results

The results of the expert panel and the Delphi study are presented separately in the following sections.

### Expert panel

The participants of the expert panel included nine people (1 female, 8 males) with a mean age of 47.5 ± 12 years and 18 ± 12 years of work experience. The proposed telehealth business framework was presented in the expert panel and based on the experts’ opinions; some components of the framework, such as process reengineering and selling licenses were removed. However, the experts suggested adding new components, such as partners’ expertise, required capital and financial resources, research and analysis, marketing and branding, tax, product registration, and marketing at scientific congresses and science and technology exhibitions. Then, the framework was validated by more experts in a Delphi study.

### Delphi study

The demographic characteristics of the participants in the first, second, and third rounds of the Delphi study are presented in Table 1.

**Table 1:** Participants’ characteristics in the first, second and third rounds of the Delphi study

Variables	Round one	Round two	Round three
	Frequency (%)	Frequency (%)	Frequency (%)
Sex			
Male	12 (57.1)	10 (71.4)	10 (71.4)
Female	9 (2.9)	4 (28.6)	4 (28.6)
Age (years)			
30–40	6 (28.6)	4 (28.6)	4 (28.6)
41–50	7 (33.3)	3 (21.4)	3 (21.4)
51–60	7 (33.3)	7 (50)	7 (50)
61–70	1 (4.8)	0	0
Education			
Ph.D.	18 (85.7)	12 (85.7)	12 (85.7)
M.Sc.	3 (14.3)	2 (14.3)	2 (14.3)
Job			
Faculty member	17 (81)	12 (85.7)	12 (85.7)
Start-up manager	4 (19)	2 (14.3)	2 (14.3)
Work experience (years)			
≤ 15	8 (38)	5 (35.7)	5 (35.7)
15 <	13 (62)	9 (64.3)	9 (64.3)

As Table 1 shows, in the Delphi study, a majority of the participants were male, had at least 15 years of work experience, and were university faculty members.

### Round one

According to the results of the first round of the Delphi study (Table 2), in the first dimension of the framework which was related to the telehealth business prerequisites, the highest mean value was related to determining the required capital and financial resources (4.75 ± 0.50), and the lowest mean value belonged to the trust in the idea registration centers (4.04 ± 0.92). In this dimension, nine components reached a consensus, and two components; namely, trust in the idea registration centers (n = 15, 71.4%) and the time lag between the idea generation and manufacturing (n = 15, 71.4%) did not reach a consensus, and were asked again the second round of the Delphi study.

**Table 2:** Participant’s responses about the importance of the components in telehealth business prerequisites and production dimensions (Round one)

No	Dimensions	Components	Subcomponents	Very important	Important	Neutral	Less important	Unimportant	Mean ± SD	Median (1st–3rd quartile)	Agreement
				Frequency (%)	Frequency (%)	Frequency (%)	Frequency (%)	Frequency (%)			
1	Telehealth business prerequisites	Value proposition	Financial value	11 (52.4)	5 (23.8)	5 (23.8)	0	0	4.28 ± 0.84	5 (4–5)	
2			Non-financial value	13 (61.9)	6 (28.6)	1 (4.8)	1 (4.8)	0	4.47 ± 0.81	5 (4–5)	✓
3			Value capture	11 (52.4)	6 (28.6)	4 (19)	0	0	4.33 ± 0.79	5 (4–5)	✓
4			Delivering value	7 (33.3)	11 (52.4)	2 (9.5)	1 (4.8)	0	4.14 ± 0.79	4 (4–5)	✓
5		Business initial requirements	Intellectual property rights	11 (52.4)	7 (33.3)	3 (14.3)	0	0	4.38 ± 0.74	5 (4–5)	✓
6			Trust in idea registration centers	8 (38.1)	7 (33.3)	5 (23.8)	1 (4.8)	0	4.04 ± 0.92	4 (3–5)	*
7			Time lag between the idea generation and manufacturing	11 (52.4)	4 (19)	4 (19)	2 (9.5)	0	4.14 ± 1/06	5 (3–5)	*
8			Team working skills	16 (76.2)	5 (23.8)	0	0	0	4.67 ± 0.43	5 (5–5)	✓
9			Business plan	11 (52.4)	9 (42.9)	1 (4.8)	0	0	4.47 ± 0.60	5 (4–5)	✓
10			Partners’ expertise	12 (57.1)	9 (42.9)	0	0	0	4.57 ± 0.50	5 (4–5)	✓
11			Required capital and financial resources	12 (57.1)	9 (42.9)	0	0	0	4.75 ± 0.50	5 (4–5)	✓
12	Telehealth business production	Key resources	Human resources	18 (85.7)	3 (14.3)	0	0	0	4.85 ± 0.35	5 (5–5)	✓
13			Physical resources	6 (28.6)	11 (52.4)	4 (19)	0	0	4.09 ± 0.70	4 (4–5)	✓
14			Financial resources	11 (52.4)	7 (33.3)	3 (14.3)	0	0	4.38 ± 0.74	5 (4–5)	✓
15		Key activities	Multi-stage assessment of the product	11 (52.4)	9 (42.9)	1 (4.8)	0	0	4.47 ± 0.60	5 (4–5)	✓
16			Research and analysis	16 (76.2)	4 (19)	1 (4.8)	0	0	4.71 ± 0.56	5 (5–5)	✓
17			Marketing and branding	12 (57.1)	9 (42.9)	0	0	0	4.57 ± 0.50	5 (4–5)	✓
18			Business counselling and mentorship	8 (38.1)	12 (57.1)	1 (4.8)	0	0	4.33 ± 0.57	4 (4–5)	✓
19			Effective communication with the stakeholders	8 (38.1)	11 (52.4)	2 (9.5)	0	0	4.28 ± 0.64	4 (4–5)	✓
20			Risk management (e.g., clinical, legal, and market risks)	11 (52.4)	8 (38.1)	2 (9.5)	0	0	4.42 ± 0.67	5 (4–5)	✓
21	Telehealth business production	Key partners	Legal partners (Public and private companies)	5 (23.8)	14 (66.7)	1 (4.8)	1 (4.8)	0	4.09 ± 0.70	4 (4–4)	✓
22			Real partners (e.g., physician, patients, treatment staff)	12 (57.1)	8 (38.1)	1 (4.8)	0	0	4.52 ± 0.60	5 (4–5)	✓
23			NGOs’ partnership	4 (19)	6 (28.6)	10 (47.6)	0	1 (4.8)	3.57 ± 0.97	3 (3–4)	*
24		Licenses and permissions	Security-based licenses	14 (66.7)	5 (23.8)	2 (9.5)	0	0	4.57 ± 0.67	5 (4–5)	✓
25			General and optional licenses	3 (14.3)	12 (57.1)	3 (14.3)	1 (4.8)	2 (9/5)	3.61 ± 1.11	4 (3–4)	*
26			Legislations in all areas	13 (61.9)	4 (19)	2 (9.5)	1 (4.8)	1 (4.8)	4.28 ± 1.14	5 (4–5)	✓
27		Stakeholders	Partnership with stakeholders	12 (57.1)	8 (38.1)	1 (4.8)	0	0	4.52 ± 0.60	5 (4–5)	✓
28			Discussions between the stakeholders	7 (33.3)	10 (47.6)	3 (14.3)	0	0	4.04 ± 0.97	4 (4–5)	✓
29			Stakeholder credibility	9 (42.9)	10 (47.6)	2 (9.5)	0	0	4.33 ± 0.65	4 (4–5)	✓
30		Market	Current and future competitors	11 (52.4)	7 (33.3)	3 (14.3)	0	0	4.38 ± 0.74	5 (4–5)	✓
31			Structure of the market	8 (38.1)	8 (38.1)	5 (23.8)	0	0	4.14 ± 0.79	4 (4–5)	✓
32			Marketing and supporting strategies	12 (57.1)	8 (38.1)	1 (4.8)	0	0	4.52 ± 0.60	5 (4–5)	✓
33			Competitiveness in the market	15 (71.4)	6 (28.6)	0	0	0	4.71 ± 0.46	5 (4–5)	✓
34		Support services	Financial support	10 (47.6)	8 (38.1)	3 (14.3)	0	0	4.33 ± 0.73	4 (4–5)	✓
35			Legal and policy protections	10 (47.6)	8 (38.1)	2 (9.5)	1 (4.8)	0	4.23 ± 0.99	4 (4–5)	✓
36			Customer and user support	16 (76.2)	5 (23.8)	0	0	0	4.76 ± 0.43	5 (5–5)	✓
37			Insurance companies’ support	13 (61.9)	5 (23.8)	2 (9.5)	1 (4.8)	0	4.42 ± 0.87	5 (4–5)	✓
38			Supporting manufacturers	10 (47.6)	8 (38.1)	2 (9.5)	0	0	4.23 ± 0.99	4 (4–5)	✓
39			Technical services (appropriate technical infrastructure, data exchange standards, confidentiality protocols, etc.)	16 (76.2)	5 (23.8)	0	0	0	4.76 ± 0.43	5 (5–5)	✓
40	Telehealth business production	Customers and users	Demand-based production	11 (52.4)	10 (47.6)	0	0	0	4.52 ± 0.51	5 (4–5)	✓
41			User interface design	12 (57.1)	8 (38.1)	1 (4.8)	0	0	4.52 ± 0.60	5 (4–5)	✓
42			Customer relationship management	13 (61.9)	8 (38.1)	0	0	0	4.61 ± 0.49	5 (4–5)	✓
43			User training	10 (47.6)	11 (52.4)	0	0	0	4.47 ± 0.51	5 (4–5)	✓
44			Healthcare delivery models	11 (52.4)	9 (42.8)	1 (4.8)	0	0	4.47 ± 0.60	5 (4–5)	✓
45			User acceptance	11 (52.4)	9 (42.8)	1 (4.8)	0	0	4.47 ± 0.60	5 (4–5)	✓
46	Telehealth business payments and costs	Cost structure	Production and logistics costs	6 (28.6)	12 (57.1)	3 (14.3)	0	0	4.14 ± 0.65	4 (5–4)	✓
47			Cost of hardware and software infrastructure	10 (47.6)	9 (42.8)	1 (4.8)	1 (4.8)	0	4.33 ± 0.79	4 (5–4)	✓
48			Cost of commercialization	11 (52.4)	7 (33.3)	3 (14.3)	0	0	4.38 ± 0.74	5 (5–4)	✓
49			Tax	6 (28.6)	4 (19)	8 (38.1)	3 (14.3)	0	3.61 ± 1.07	3 (3–5)	*
50			Other tangible and intangible costs	6 (28.6)	5 (23.8)	10 (47.6)	0	0	3.80 ± 0.87	4 (5–3)	*
51		Pricing	Pricing by product manufacturers	5 (23.8)	8 (38.1)	8 (38.1)	0	0	3.85 ± 0.79	4 (3–4)	*
52			Pricing by an independent organization	4 (19)	7 (33.3)	7 (33.3)	3 (14.3)	0	3.57 ± 0.97	4 (3–4)	*
53			Economical pricing	11 (52.4)	7 (33.3)	2 (9.5)	1 (4.8)	0	4.33 ± 0.65	4 (4–5)	✓
54		Revenue making	Selling products	8 (38.1)	8 (38.1)	4 (19)	0	1 (4.8)	4.04 ± 1.02	4 (5–4)	✓
55			Selling data	4 (19)	7 (33.3)	7 (33.3)	2 (9.5)	1 (4.8)	3.52 ± 1.07	4 (3–4)	*
56			Receiving payments from product users	7 (33.3)	11 (52.4)	3 (14.3)	0	0	4.19 ± 0.67	4 (5–4)	✓
57		Revenue model	Revenue making strategies	10 (47.6)	9 (42.8)	1 (4.8)	0	1 (4.8)	4.28 ± 0.95	4 (4–4)	✓
58			Current and future revenue streams	6 (28.6)	11 (52.4)	3 (14.3)	0	1 (4.8)	4 ± 0.94	4 (5–4)	✓
59			Financial stability	14 (66.6)	6 (28.6)	1 (4.8)	0	0	4.61 ± 0.58	5 (5–4)	✓
60			Profitability	11 (52.4)	7 (33.3)	1 (4.8)	1 (4.8)	1 (4.8)	4.23 ± 1.09	5 (5–4)	✓
61	Telehealth business post-production services	Product evaluation	In-person feedback	7 (33.3)	8 (38.1)	4 (19)	1 (4.8)	1 (4.8)	3.90 ± 1.09	4 (5–3)	*
62			Electronic feedback	7 (33.3)	11 (52.4)	3 (14.3)	0	0	3.61 ± 1.11	4 (3–4)	✓
63			Research-based feedback	8 (38.1)	9 (42.8)	4 (19)	0	0	4.19 ± 0.74	4 (5–4)	✓
64		Responsibility of telehealth services	Full responsibility for providing telehealth services	12 (57.1)	8 (38.1)	0	0	1 (4.8)	4.47 ± 0.74	5 (5–4)	✓
65			Relative responsibility for providing telehealth services	3 (14.3)	10 (47.6)	7 (33.3)	1 (4.8)	0	4.04 ± 0.97	4 (4–5)	*
66		Product Protection	Patent registration	5 (23.8)	8 (38.1)	4 (19)	4 (19)	0	3.57 ± 1.24	4 (3–4)	*
67			Maintaining confidentiality in telehealth services	12 (57.1)	5 (23.8)	3 (14.3)	1 (4.8)	0	4.33 ± 0.91	5 (5–4)	✓
68			Official product registration	10 (47.6)	9 (42.8)	2 (9.5)	0	0	4.33 ± 0.66	4 (5–4)	✓
69			Receiving certificate of excellence	8 (38.1)	7 (33.3)	2 (9.5)	2 (9.5)	2 (9.5)	3.80 ± 1.32	4 (5–3)	*
70		Product marketing	Traditional marketing	14 (66.7)	4 (19)	3 (14.3)	0	0	4.52 ± 0.74	4 (5–4)	✓
71			Digital marketing	9 (42.8)	9 (42.8)	3 (14.3)	0	0	4.28 ± 0.71	4 (5–4)	✓
72			Marketing at scientific congresses and science and technology exhibitions	9 (42.8)	5 (23.8)	4 (19)	2 (9.5)	1 (4.8)	3.90 ± 1.22	4 (5–3)	*
73		Making contracts	Commercial contracts	11 (52.4)	9 (42.8)	1 (4.8)	0	0	4.47 ± 0.60	5 (5–4)	✓
74			Non-commercial contracts	4 (19)	9 (42.8)	5 (23.8)	2 (9.5)	1 (4.8)	3.61 ± 1.07	4 (3–4)	*

Agreement ✓ No agreement *

Regarding the production dimension, the results indicated that most components were either very important or important from the participants’ perspectives. In this dimension, the highest mean value (4.85 ± 0.35) was related to human resources under the key resources component and the lowest mean value (3.57 ± 0.97) belonged to the NGOs’ partnerships under the key partners component. As less than 50% of the participants (n = 10, 47.6%) agreed on the importance of NGOs’ partnership, this item was removed from the final framework. In addition, one component; namely, general and optional licenses under the licenses and permissions component (n = 15, 71.4%) did not reach a consensus, and was included in the second round of the Delphi study.

Among the components of the payments and costs dimension, financial stability (4.61 ± 0.58) and selling data (3.52 ± 1.07) had the highest and lowest mean values, respectively. In this dimension, less than 50% of the participants agreed on the importance of tax (n = 10, 47.6%). As a result, this component was removed from the framework. Four other components; namely, other tangible and intangible costs (n = 11, 52.4%), pricing by product manufacturers (n = 13, 61.9%), pricing by an independent organization (n = 11, 52.4%), and selling data (n = 11, 52.4%) did not reach a consensus and were asked again in the second round of the Delphi study.

The fourth dimension of the framework was related to the post-production services. The results showed that the highest mean value belonged to the traditional marketing (4.52 ± 0.74) and the lowest mean value was related to patent registration (3.57 ± 1.24). While most of the components of this dimension were found important by the participants, in-person feedback (n = 15, 71.4%), relative responsibility for providing telehealth services (n = 13, 61.9%), patent registration (n = 13, 61.9%), receiving certificate of excellence (n = 15, 71.4%), marketing at scientific congresses as well as science and technology exhibitions (n = 14, 66.7%), and non-commercial contracts (n = 13, 61.9%), did not reach a consensus and included in the second round of the Delphi study.

### Round two

As Table 3 shows, the time lag between the idea generation and manufacturing (4.78 ± 0.42) had the highest mean value and non-commercial contracts had the lowest mean value (3.64 ± 0.74) in the second round of the Delphi study. As trust in idea registration centers (n = 9, 64.3%), pricing by an independent organization (n = 9, 64.3%), in-person feedback (n = 10, 71.4%), patent registration (n = 9, 64.3%), and non-commercial contracts (n = 9, 64.3%) did not reach a consensus, they were asked again in the third round of the Delphi study.

**Table 3:** Participant’s responses about the importance of the components of a telehealth business framework (Round two of the Delphi study)

No	Dimensions	Components	Subcomponents	Very important	Important	Neutral	Less important	Unimportant	Mean ± SD	Median (1st–3rd quartile)	Agreement
				Frequency (%)	Frequency (%)	Frequency (%)	Frequency (%)	Frequency (%)			
1	Telehealth business prerequisites	Business initial requirements	Trust in idea registration centers	4 (28.6)	5 (35.7)	4 (28.6)	1 (7.1)	0	3.85 ± 0.94	4 (3–4.75)	*
2			Time lag between the idea generation and manufacturing	11 (78.6)	3 (21.4)	0	0	0	4.78 ± 0.42	5 (5–5)	✓
3	Telehealth business production	Licenses and permissions	General and optional licenses	6 (42.9)	8 (57.1)	0	0	0	4.42 ± 0.51	4 (4–5)	✓
4	Telehealth business payments and costs	Cost structure	Other tangible and intangible costs	9 (64.3)	2 (14.3)	3 (21.4)	0	0	4.42 ± 0.85	5 (4–5)	✓
5		Pricing	Pricing by product manufacturer	4 (28.6)	8 (57.1)	2 (14.3)	0	0	4.14 ± 0.66	4 (4–4.75)	✓
6			Pricing by an independent organization	3 (21.4)	6 (42.9)	4 (28.6)	1 (7.1)	0	3.78 ± 0.89	4 (3–4)	*
7		Revenue making	Selling data	4 (28.6)	8 (57.1)	1 (7.1)	1 (7.1)	0	4.07 ± 0.82	4 (4–4.75)	✓
8	Telehealth business post-production services	Product evaluation	In-person feedback	7 (50)	3 (21.4)	3 (21.4)	0	1 (7.1)	4.07 ± 1.20	4.5 (3.5–4.5)	*
9		Responsibility of telehealth services	Relative responsibility for providing telehealth services	5 (35.7)	7 (50)	2 (14.3)	0	0	4.21 ± 0.69	4 (4–5)	✓
10		Product Protection	Patent registration	3 (21.4)	6 (42.9)	4 (28.6)	1 (7.1)	0	3.78 ± 0.89	4 (3–4)	*
11			Receiving certificate of excellence	6 (42.9)	5 (35.7)	2 (14.3)	1 (7.1)	0	4.14 ± 0.94	4 (4–5)	✓
12		Product marketing	Marketing at scientific congresses and science and technology exhibitions	7 (50)	3 (21.4)	3 (21.4)	1 (7.1)	0	4.14 ± 1.02	4.5 (3.5–4.5)	✓
13		Making contracts	Non-commercial contracts	1 (7.1)	8 (57.1)	4 (28.6)	1 (7.1)	0	3.64 ± 0.74	4 (3–4)	*

Agreement ✓ No agreement *

### Round three

Table 4 shows the results of the third round of the Delphi study. In this round only one component; namely, in-person feedback (n = 11, 78.6%) reached a consensus. Other items including trust in idea registration centers (n = 10, 71.4%), pricing by an independent organization (n = 9, 64.3%), patent registration (n = 10, 71.4%), and non-commercial contracts (n = 10, 71.4%) did not reach a consensus and were removed from the final framework.

**Table 4:** Participant’s responses about the importance of the components of a telehealth business framework (Round three of the Delphi study)

No	Dimensions	Components	Subcomponents	Very important	Important	Neutral	Less Important	Unimportant	Mean ± SD	Median (1st–3rd quartile)	Agreement
				Frequency (%)	Frequency (%)	Frequency (%)	Frequency (%)	Frequency (%)			
1	Telehealth business prerequisites	Business initial requirements	Trust in idea registration centers	2 (14.3)	8 (57.1)	3 (21.4)	1 (7.1)	0	3.74 ± 0.80	4 (3.25–4)	*
2	Telehealth business payments and costs	Pricing	Pricing by an independent organization	4 (28.6)	5 (35.7)	3 (21.4)	2 (14.3)	0	3.78 ± 1.05	4 (3–4.75)	*
3	Telehealth business post-production services	Product evaluation	In-person feedback	6 (42.9)	5 (35.7)	2 (14.3)	0	1 (7.1)	4.07 ± 1.14	4 (4–5)	✓
4		Product Protection	Patent registration	2 (14.3)	8 (57.1)	3 (21.4)	0	1 (7.1)	3.71 ± 0.99	4 (3.25–4)	*
5		Making contracts	Non-commercial contracts	0	10 (71.4)	4 (28.6)	0	0	3.71 ± 0.46	4 (3.25–4)	*

Agreement ✓ No agreement *

**Fig. 1:**
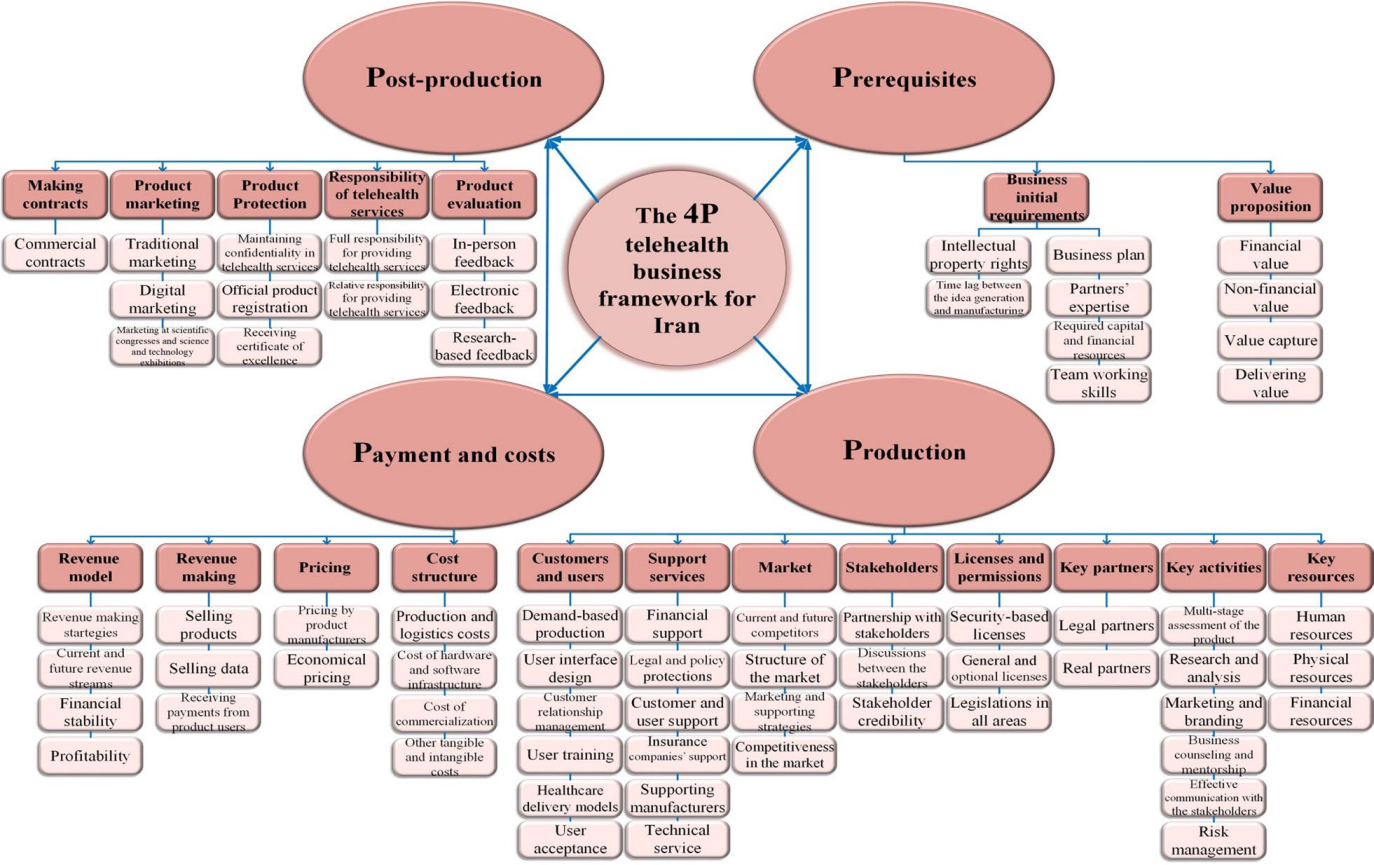
The 4P telehealth business framework for Iran

Finally, after three rounds of the Delphi study, six out of 74 components were removed, leaving 68 components in four dimensions. As all dimensions started with “P” letter, the final framework was named “the 4P telehealth business framework” (Fig. 1).

## Discussion

Business models and frameworks have been used in various fields including information technology and management sciences and telehealth technology. [28–30]. However, various definitions and components have been presented for them and the field of telehealth included a limited number of components [29–31]. Also, there is no unique way to identify components of business models and frameworks and they may include a combination of components [32, 33]. In the present study, the aim was to develop a telehealth business framework for Iran. The proposed framework, which was initially derived from conducting a systematic review [24] and a qualitative study [27], was reviewed by an expert panel who believed that components, such as process reengineering and selling licenses should be removed. According to the experts, the first one was beyond the business activities, and the second one needed legal support. However, they suggested adding new components, such as partners’ expertise, required capital and financial resources, research and analysis, marketing and branding, tax, product registration, and marketing at scientific congresses and science and technology exhibitions to the framework. After doing modifications, the framework was presented to a larger group of the experts who approved a majority of components after three rounds of the Delphi study.

The final framework included four main dimensions; namely telehealth business prerequisites, production, payments and costs, and post-production services which, in turn consisted of a number of components necessary for developing a telehealth business plan. This framework like other business models and frameworks can be used as a conceptual tool in systematic and holistic thinking [29, 34] to demonstrate how innovation, technology, and associated knowledge are converted into profit flow through the use of tangible and intangible assets [35]. In contrast to the previous telehealth business models and frameworks, such as VISOR business model framework [36] and CompBizMod framework [18] which have focused on a limited number of components, this study incorporated various organizational, financial, technical, and non-technical components into the telehealth business framework to present a bigger picture of the influencing components on a successful telehealth business.

According to Chen et al., business modeling is a collaborative effort to create value, in which all stakeholders influence the needs of others [22]. Nikou and Bouwman, on the other hand, concentrated on underlying health issues, such as users’ needs and experiences, and paid less attention to the non-technical components such as value proposition, organizational model, business model, and revenue models [37]. In another study, Alami et al. indicated that integrating professional, clinical, organizational, technological, and systematic aspects of telehealth is critical for developing an integrated vision. In fact, a multi-stakeholder strategy, in which professional, technological, organizational, and political perspectives are considered, is necessary for running a telehealth business. Such a strategy should be supported by evolutionary approaches, such as financial management, change management, and government approval [38]. Therefore, it seems that the framework developed in the current study has covered many aspects of a telehealth business mentioned in other similar studies.

Apart from the framework dimensions and components, many studies have highlighted the importance of using telehealth business models and frameworks. Parimbelli et al., for example, emphasized the importance of developing a transparent, predictable, and sustainable regulatory framework for the telehealth industry in order to implement innovations and ensure maintaining high standards of patient safety [39]. Furthermore, Stroetmann argued that the potentials of e-health innovations should be taken into account, and governments must provide the necessary infrastructure to deal with the failure of these technologies on the market and assist in their integration with other existing systems. To ensure the efficiency of such an investment, studies such as regulatory impact analyses (RIAs) and socioeconomic cost–benefit analyses (SCBAs) are recommended [40].

Overall, the results showed that multiple dimensions and components may influence a telehealth business, and their impact might be interrelated. Therefore, the role these components in a real telehealth business can be evaluated separately and jointly.

### Research implications

Our findings contributed to the existing literature by providing a comprehensive telehealth business framework for developing successful telehealth start-ups and businesses. The current research also supports the theoretical arguments asserting the importance of using a multi-dimension/component framework in the field of telehealth and facilitates developing telehealth business plans. It seems that sustainability in the market and potentials for becoming competitive can be improved by considering the components of the current framework in the future telehealth business plans. Moreover, this framework can be used to evaluate the current state of telehealth start-ups and improve their performance.

In terms of clinical practice, it is important to have available, valid and suitable telehealth technology for healthcare professionals and patients. As the use of telehealth technology is expanding, particularly after the Covid-19 pandemic, start-ups can use this opportunity to develop and implement new technologies. However, the sustainability and success of their products in the market may depend upon the technical and non-technical components of the current telehealth business framework. The more the framework components are considered, the higher the success of the business can be expected, which, in turn, will be followed by increasing the use of the telehealth technology by the end-users.

In terms of future research, examining efficiency and effectiveness of the current framework in different telehealth businesses and comparing the results are suggested. Moreover, this framework can be adopted in other countries and more components can be added to, or removed from it.

### Research limitations

Although the 4P telehealth business framework was developed for the first time in this study, the research had limitations. First of all, the number of the participants in the first, second, and third rounds of the Delphi study was limited. The reasons for not-taking part in the research might be related to the Covid-19 pandemic, busy schedules, and the lack of interest in the topic of the research. However, as the participants were expert in the field of telehealth business and had relevant work experience, it seems that the results are generalizable to a wider population.

The second limitation was related to the components and details of the 4P telehealth business framework. As there were too many components and subcomponents for each dimension, only the general ones were included in the framework to avoid making the framework complicated. In future research, other components can be added to, or removed from the current framework.

Moreover, this framework was developed for Iran, which may have different characteristics from other countries. While general dimensions and components of the framework can be used in other countries, some components may need to be modified to reflect each country's political, economic, and health characteristics.

## Conclusion

Business models and frameworks are a collection of critical components that facilitate the process of value creation from business ideas and using them is regarded as a competitive advantage. In the field of telehealth technology, business models and frameworks contribute significantly to the successful implementation and commercialization of the ideas and technologies in this field. These models and frameworks are still evolving, and their full potentials appear to have not been realized yet. The findings of the current study indicated that there are numerous components necessary to develop telehealth business models and frameworks. Paying adequate attention to these components as a framework can facilitate commercializing telehealth technologies and developing business plans. In addition, telehealth start-ups can use this framework to improve their success and sustainability in a competitive market. It seems that the framework proposed in the current study can also be used by other countries, as most of the dimensions, components, and subcomponents are common in telehealth businesses. However, the effectiveness of this framework in practice and in successful commercialization of telehealth technologies should be evaluated in the future research.

## Data Availability

The data used and analyzed during the current study are available from the corresponding author on reasonable request.

## Abbreviations

4P: Prerequisites, production, payments and costs, and post-production services
BM: Business model
RIA: Regulatory impact analysis
SCBA: Socioeconomic cost–benefit analysis
